# Chamber Bioaerosol Study: Outdoor Air and Human Occupants as Sources of Indoor Airborne Microbes

**DOI:** 10.1371/journal.pone.0128022

**Published:** 2015-05-29

**Authors:** Rachel I. Adams, Seema Bhangar, Wilmer Pasut, Edward A. Arens, John W. Taylor, Steven E. Lindow, William W. Nazaroff, Thomas D. Bruns

**Affiliations:** 1 Department of Plant and Microbial Biology, University of California, Berkeley, Berkeley, California, United States of America; 2 Department of Civil and Environmental Engineering, University of California, Berkeley, Berkeley, California, United States of America; 3 Center for the Built Environment, University of California, Berkeley, Berkeley, California, United States of America; Tsinghua University, CHINA

## Abstract

Human occupants are an important source of microbes in indoor environments. In this study, we used DNA sequencing of filter samples to assess the fungal and bacterial composition of air in an environmental chamber under different levels of occupancy, activity, and exposed or covered carpeting. In this office-like, mechanically ventilated environment, results showed a strong influence of outdoor-derived particles, with the indoor microbial composition tracking that of outdoor air for the 2-hour sampling periods. The number of occupants and their activity played a significant but smaller role influencing the composition of indoor bioaerosols. Human-associated taxa were observed but were not particularly abundant, except in the case of one fungus that appeared to be transported into the chamber on the clothing of a study participant. Overall, this study revealed a smaller signature of human body-associated taxa than had been expected based on recent studies of indoor microbiomes, suggesting that occupants may not exert a strong influence on bioaerosol microbial composition in a space that, like many offices, is well ventilated with air that is moderately filtered and moderately occupied.

## Introduction

Human occupants are an important source of microbes in indoor environments. On indoor surfaces, direct contact leads to a rapidly generated signature of the occupants [1], one that is predictable based on the nature of the human contact [2]. Airborne microbial levels increase when rooms are occupied compared to unoccupied conditions [3, 4], and humans have been reported to be a source of bacteria and fungi in settled dust samples [5–7].

A full understanding of the role that occupancy plays in airborne microbial quantity and composition, particularly in comparison with other sources such as ventilation supply from outdoor air, is just beginning to emerge. While a recent study showed the influence of human body-associated bacteria in office buildings [8], investigations into the source strength of humans on indoor bioaerosols have predominantly focused on occupied classrooms [9–13]. Hospodsky et al. [10] showed that human occupancy in a university classroom setting leads to a nearly 10× increase in bacterial genomes and that emissions from human skin make a significant direct contribution to the bacteria in indoor air. More recently, Hospodsky et al. [11] determined in a sample of children’s classrooms that the emission rates attributable to occupants ranged from 0.8 million to 35 million bacteria cells per person-hour and from 3 million to 57 million fungal cells per person-hour. Two broad routes have been identified through which human occupants emit bioaerosol particles. One route is through direct shedding, which includes particles directly coming off bodies and clothing. The second route is through resuspension from inanimate room surfaces, whereby occupants’ movements disturb microbial materials that had previously settled onto or colonized indoor materials.

The goal of the present study is to explore the relative contribution of occupancy compared to other sources in shaping indoor bioaerosol composition using replicated experiments conducted under controlled conditions. For one component of the study, we sought to identify how the number and activity of occupants influences the biological components of the indoor air; we report those results here. Elsewhere, we report the emission rates of fluorescent biological aerosol particles from human occupants [14].

We combine information from filter samples subjected to molecular-based analysis of DNA with the real-time size-resolved optical monitoring of total particle number concentrations. Outdoor samples were collected simultaneously and analyzed, which allows us to put the effect of occupants in the context of the contribution of outdoor air, as introduced via the ventilation system. We hypothesized that the signature of human-emitted microbes would increase with the number and activity of occupants, would be greater when the flooring was carpet that was exposed rather than covered with plastic sheeting, and would be stronger for bacteria than fungi. We expected that airborne microbial composition during low occupancy periods would be compositionally similar to that of outdoor air. We also expected that composition during high human occupancy periods would be similar across samples because of the shared signature of the human microbiome and because of the anticipated strong contribution of occupants to total airborne levels.

## Materials and Methods

### Experimental design

**Fig 1:**
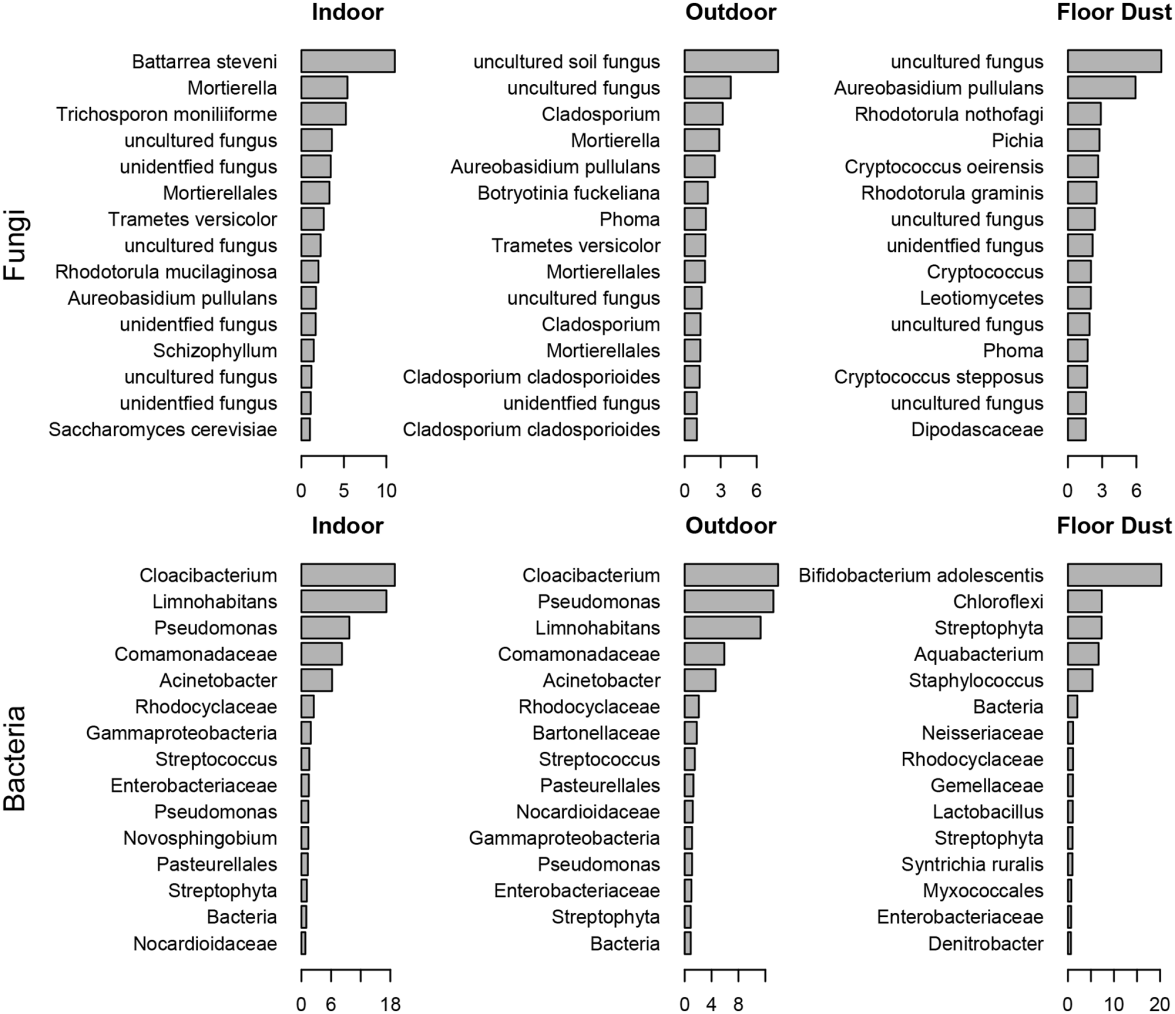
The relative abundance of the 15 most common fungal and bacterial taxa in indoor air, outdoor air, and vacuumed floor dust samples. The x-axis represents the percentage of sequences in each category (indoor, outdoor, vacuum) represented by the top taxa, which were identified to the finest taxonomic level that could be assigned.

Experiments were conducted in a controlled environmental chamber (Fig 1) designed to simulate an office room [15]. The chamber has a floor area of 30 m^2^ and the ceiling height is 2.5 m. Ventilation in the chamber is controlled by an independent heating, ventilation, and air-conditioning (HVAC) system. The HVAC system supplies thermally and humidity controlled outdoor air that has passed through filters with a MERV 7 rating. There is no air recirculation with the single-pass ventilation system. The duct length from the outside air inlet to the room is approximately 10 m, and the ventilation air passes through a filter, supply fan, cooling coil, and heating coil in sequence. Outdoor temperatures during the test period ranged from 10 to 20°C. The outdoor humidity conditions were such that the cooling coil temperature was always above the dewpoint and therefore dry. The room was pressurized so that infiltration from adjacent building spaces was negligible. The ventilation fan speed was set to maintain a constant air-exchange rate, which was calculated by the exponential decay of the net CO_2_ level (indoor minus outdoor) during the post-experimental period when the room quickly went from being occupied to unoccupied. The air-exchange rate was so determined to be 2.8 ± 0.2 h^-1^.

Experimental treatments varied by the number of people in the room (0, 1, 2, or 8 people) and their activity (sitting versus walking, the latter used only for the 2-occupant case). The chamber floor consisted of closed-loop nylon carpet tiles, and we executed experiments both with the chamber carpet exposed and with it covered in plastic sheeting. To minimize static electricity effects during the walking treatment of the covered floor, we placed rectangular strips of a conductive material on top of the plastic sheeting. Subjects walked only on these strips. The duration of each experiment was two hours, a period of time allowing for sufficient collection of bioaerosol material for community analysis while still a time-resolved sampling period aligned with the particle-based instrumentation The five experimental combinations of occupancy and activity were each replicated three times on two different floor types, for a total of 30 experiments (S1 Table).

Participants were members of the university community (age 18+), including students, researchers, and faculty, and each of the high-occupancy sampling periods included both men and women [14]. Participants provided written informed consent for the research, which was approved by the University of California Committee for the Protection of Human Subjects Protocol ID 2013-01-4927.

### Sample collection

Bioaerosol samples were collected by drawing air through open-face filters. Analytic filter cups of 47 mm diameter, 0.2 μm pore size cellulose nitrate membrane (Thermo Scientific # 145–0020, purchased from Fisher Scientific, Chicago, IL, USA) were suspended upside down at a height of 1.5 m (S1 Fig). The attached vacuum pump (model #0523-101Q-sg588DX, Gast Manufacturing Inc, Benton Harbor, MI, USA) was set to a flow rate of 25 liters per minute, so that a total volume of 3 m^3^ of air was sampled per experiment. We note that in sampling the entire 2-hour experimental period, the procedure captures both times when conditions are in transition and when steady-state conditions should prevail. For outdoor samples, the filter cup was suspended upside down, outside a window on the same building face at ~ 5 m from the outdoor air intake for the HVAC system. This sampling approach should only collect particles with diameters smaller than ~ 50 μm (assuming spherical particles with density 2.5 g/cm^3^). The settling velocities of larger particles would exceed the upward speed of air entering the filter cups.

Two vacuumed samples of floor dust from the chamber carpet were collected at the beginning and end of the study period, December 2013. The Dustream Collector (Indoor Biotechnologies, Charlottesville, NC, USA) was used to isolate the vacuumed material in the head of the sampling wand [16], and the vacuum cleaner was run for a 1-min duration while moving across the full extent of the chamber floor.

Total particle number concentrations were measured at a frequency of once per minute with the Met One GT 526 optical particle counters (OPCs; Grants Pass, OR, USA), which measures particle number concentrations in six bins according to optical diameter: 0.3–0.5, 0.5–0.7, 0.7–1.0, 1.0–2.0, 2.0–5, and >5 μm, respectively.

### Molecular analysis

DNA extraction protocols followed those used previously for indoor bioaerosols [17] and are detailed in S1 File. Starting material was half of the filter from the filter cup or 200 mg of unprocessed dust from the floor dust sample. To determine the composition of the microbial communities, we used the approach of sequencing a universal “barcode,” i.e., a region of DNA targeted to a specific group of organisms that can be used for identification in samples containing a mixture of many taxa [18]. Specifically, we targeted the V4-V5 region of the bacterial 16S rRNA gene and the ITS1 region of the fungal rRNA gene. Bacterial primers were those adopted for the Earth Microbiome Project [19] while fungal primers were those recently described by Smith and Peay [20]. Samples were split across two Illumina MiSeq lanes for 250 base pair paired-end sequencing at the Stanford Functional Genomics Facility. The raw sequence data were deposited into NCBI’s Sequence Read Archive (SRA) under study accession SRP049464.

### Bioinformatic processing and data analysis

For fungi both the forward (“R1”) and reverse (“R2”) reads for each sequence could be paired before downstream analysis. For bacteria the quality of the R2 sequencing reads was low; consequently we only proceeded with the R1 reads. The general processing approach involved quality filtering, pairing reads (for fungi), clustering reads into operational taxonomic units (OTUs) at 97% similarity, checking for chimeric sequences, and identifying taxonomy against a reference database. To implement these steps, we utilized cutadapt [21], Trimmomatic [22], UPARSE scripts [23], homerTools [24], and the UNITE [25] and Greengenes [26] databases for fungi and bacteria, respectively. Specific program settings are detailed in S1 File.

We adopted several OTU quality filtering and benchmarking steps. First, we removed the OTUs that were unclassified after taxonomic identification. Second, we analyzed a fungal mock community of 18 fungal taxa whose abundance of extracted DNA was skewed to mimic a natural community and then pooled. Examining the sequences of the two mock community samples, we found that only taxa with 10 or more sequences should be included in further analyses. That is, the mock community was recovered only when OTUs with greater than 10 reads were considered; otherwise, the mock community had much higher richness than initially pooled. The specific threshold value is likely to be run-specific, as a similar approach using a mock community in another study informed a lower threshold value of 3 reads [27]. As a comparison, other studies have taken the approach of excluding sequences that do not surpass a certain percentage of all reads (e.g. [28]). Third, we processed negative extraction controls with our samples. Even though visualization of the amplicons on an agarose gel showed no amplification, sequencing yielded reads in these negative samples. We subtracted the number of sequence reads in the negative samples from the environmental samples. We note that doing analysis without these quality-filtering steps produced qualitatively similar results as those reported here. After bioinformatics processing, 3.6 million and 4.2 million fungal and bacterial sequencing reads, respectively, were retained for analysis. Analyses were executed in R [29], utilizing the vegan and labdsv packages as needed. Phylogenetic analysis of the bacteria samples was conducted in QIIME [30]. To ensure even representation of sequences per samples, samples were rarified to 5,000 sequences per sample for both fungi and bacteria.

### Supplemental experiments to sample the supply air

Initial exploration of the results suggested a higher than expected contribution of the ventilation supply air to the indoor bioaerosol composition. To assess whether the ventilation system itself might be contributing, we conducted two additional experiments in June, 2014, one with 2 people walking and one with the chamber unoccupied (S1 Table). In addition to two samples of vacuumed floor dust collected at the start and the end of the day and the outdoor and indoor air samples, we included a third air sampler deploying an analytical filter cup within the subfloor plenum, from which the supply air enters the chamber. These eight samples (two of vacuumed floor dust plus 2 × 3 of filtered air) were processed as detailed above.

## Results

Overall, short-term sampling of bioaerosols showed that the microbial communities in the chamber air were taxonomically rich and temporally variable. The microbial communities observed in the indoor air samples largely tracked those simultaneously measured outdoors, and taxa known to be associated with the human body played a secondary although important role. The behavior of particles as measured by the OPCs corroborate that the ventilation and filtration conditions of this chamber yielded strong influence from outdoor air. The following subsections provide details that elaborate on these summary findings.

### Microbial composition

Looking broadly at the composition of the identified microbes in aerosol particles, many of the common fungal taxa were familiar from culture and microscopy-based work [31], including species of *Cladosporium*, *Aureobasidium*, *Phom*a, *Alternaria*, *Rhodotorula*, and *Penicillium*. Other abundant taxa included yeasts (*Cryptococcus sp*., *Candida sp*.), plant pathogens (e.g. *Botryotinia fuckeliana*), and wood rot fungi (e.g. *Trametes versicolor*, *Stereum sp*.). The dominant bacterial phylum was *Proteobacteria*, followed by *Firmicutes* and *Actinobacteria*. In addition to the common inhabitants of human skin, gut, and oral cavities such as *Actinomycetales* (including the *Corynebacteriaceae*), *Lactobacillales* (the *Streptococcaceae*), and *Enterobacteriales*, we also observed high abundance of the outdoor-associated taxa such as *Burkholderiales*, *Pseudomonadales*, *Flavobacteriales*, and *Streptophyta* (chloroplasts). Like other environmental surveys, most taxa appeared sporadically: over 50% of the taxa were present in only one sampling period, and over 80% of the taxa appeared in less than 10% of the samples.

Considering both frequency and abundance, there was large overlap in microbial taxa between indoor and outdoor air samples. Table 1 shows the most frequently encountered taxa in the chamber air (that is, the taxa that appear in the greatest number of samples), and their corresponding frequency in outdoor samples. There were no frequently observed taxa in indoor air that were entirely absent from outdoor air. Two of the 40 taxa (5%) in this table had a greater than 2× frequency of occurrence in indoor air compared to outdoor air: the fungus *Sordaria sp*. and the bacterium *Streptococcus sp*. Fig 1 shows the abundant taxa (those with the highest number of sequencing reads) in chamber air, outdoor air, and floor dust. Only five of the 15 fungal taxa (33%) in this set were shared across the abundant indoor and outdoor air samples, whereas 12 bacterial taxa (80%) were shared. The most abundant fungal taxon indoors was *Battarea steveni*, a puffball that is discussed later in the context of human-mediated transport.

**Table 1:** Most frequently encountered taxa.

Fungi	Indoors (n = 31)	Outdoors (n = 32)
*Trametes versicolor*	87	78
*unidentfied fungus*	77	81
*Cladosporium*	77	69
*uncultured soil fungus*	74	94
*Cladosporium*	74	91
*Cladosporium dominicanum*	74	81
*Penicillium*	74	81
*Cladosporium cladosporioides*	55	72
*Phlebia tremellosa*	52	31
*Trametes*	48	53
*Wallemia*	48	31
*Sistotremastrum*	45	38
*uncultured fungus*	42	75
*Stereum*	42	72
*Eurotium*	42	59
*Cladosporium cladosporioides*	42	44
*Ceriporia viridans*	42	34
*Phlebia*	42	34
*Wallemia sebi*	42	31
*Sordaria*	42	6
**Bacteria**		
*Comamonadaceae*	90	78
*Cloacibacterium*	87	78
*Limnohabitans*	87	63
*Acinetobacter*	84	63
*Pseudomonas*	81	63
*Nocardioidaceae*	81	59
*Gammaproteobacteria*	81	59
*Rhodocyclaceae*	77	66
*Rickettsiales mitochondria*	77	66
*Streptococcus*	77	38
*Bacteria*	74	69
*Weeksellaceae*	74	63
*Brucellacea*	74	59
*Staphylococcus*	71	72
*Dorea formicigenerans*	71	56
*Afifella*	71	53
*Novosphingobium*	71	44
*Corynebacterium*	68	78
*Streptophyta*	68	75
*Enterobacteriaceae*	68	59

Percentages of samples in which the most frequently encountered taxa appear.

**Fig 2:**
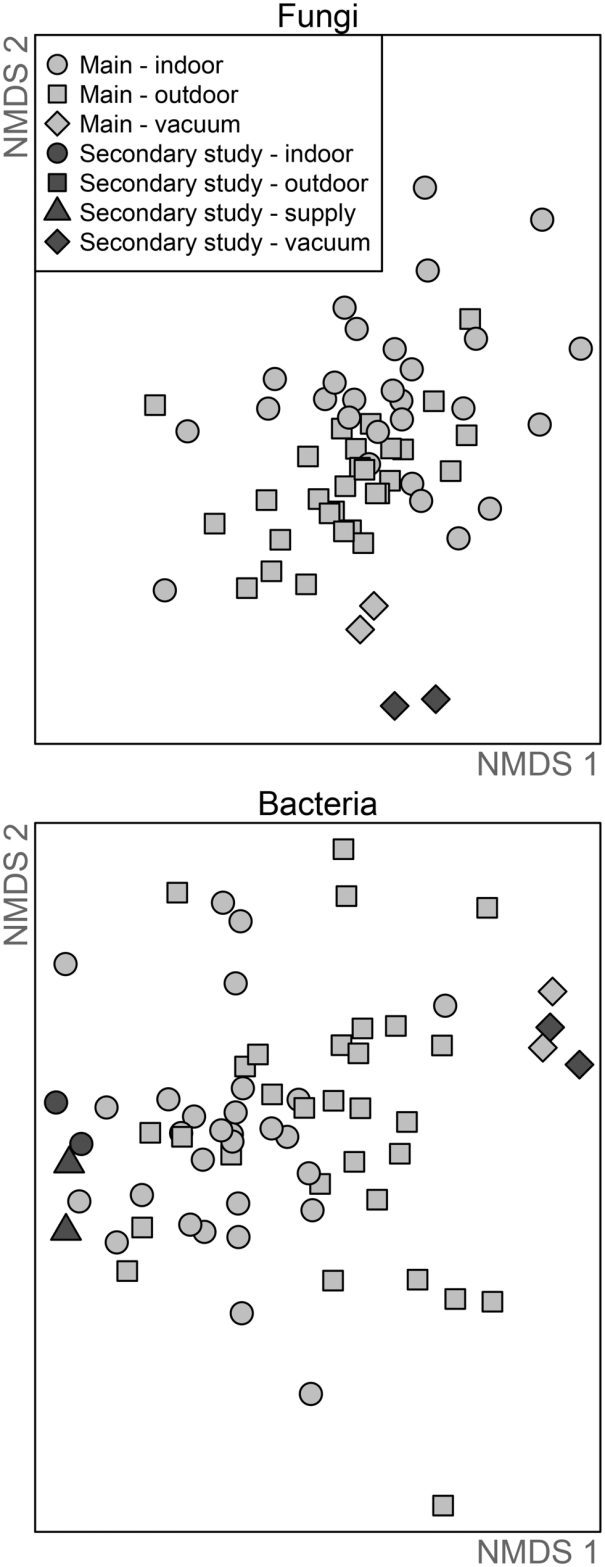
Visualization of the composition of fungal and bacterial communities across different samples. Each point represents the composition in the sample, such that those samples that are closer together share more taxa in common than those farther apart. Samples collected during the main study (light gray) are differently shaded than those from the secondary study that included sampling of the supply air (dark gray).

To determine which measured factors predict indoor microbial composition we applied an analysis of variance statistical model based on distance matrices. As shown using the Canberra community distance, fungal and bacterial communities shared similar patterns with some notable differences (Fig 2). Indoor and outdoor air samples from the main experiments were more similar to each other and distinct from samples collected during the secondary experiments, which included supply-air sampling. Within the main study period, the indoor and outdoor fungal and bacterial aerosols were significantly different from each other, both when the carpet was exposed and when it was covered (adonis *p*-value = 0.001), although the percentage of the variation in composition explained by location was marginal (*R* ^2^ = 0.04 for both fungi and bacteria). Bacterial community relationships across samples based on UniFrac, a distance index that considers phylogenetic relationships [32], yielded similar results to those based on taxonomic relationships (S2 Fig). Due to the highly variable nature of the ITS marker, phylogenetic analysis was not applied to the fungal communities.

### Effect of occupancy on bioaerosols

We applied a statistical model to determine which measured factors predict indoor microbial composition. The sampling date and occupancy level were statistically significant predictors, explaining approximately 36% and 13% of the variation in composition, respectively (Table 2). The influence of sampling date and occupancy level can be interpreted as follows: bioaerosols tend to be more similar in composition if they were collected on the same day or during the same occupancy level. After explaining variation by date and occupancy level, the effect of flooring and time of day were not significant predictors. Overall, 50% of the variation in microbial composition was unexplained by the measured variables.

**Table 2:** Factors influencing microbial composition.

	Fungi		Bacteria	
	*R* ^2^	*P*-value	*R* ^2^	*P*-value
Sampling date	0.36	**0.001**	0.37	**0.003**
Occupancy level	0.14	**0.011**	0.13	**0.031**
Time of day	0.09	0.885	0.06	0.741
Floor covering	0.03	0.876	0.04	0.869

Variance in biological dissimilarity among fungal and bacterial communities explained by different measured variables.

Higher occupancy periods were associated with ~ 2× greater taxon richness for both fungi and bacteria, and, as would be expected, this trend was unmatched in the outdoor samples (ANOVA, *p*>0.05; S3 Fig). This increase in richness did not appear to be due to the addition of human-associated taxa, as those taxa did not dominate the occupied periods when looking at the entire microbial community. Considering sequence read abundance (as in Fig 1), the sum of five human skin taxa—*Propionibacterineae*, *Staphylococcus*, *Enterobacteriaceae*, *Corynebacterineae*, and *Streptomycetaceae*—comprise 4.3% of the indoor air sequences and 3.7% of the outdoor air samples, indicating only a modest enrichment of these species as contributors to indoor air microbial composition. Considering the frequency of taxon occurrence (as in Table 1), there were 26 fungal taxa found in at least five of the six 8-person experiments, and only two (8%) showed increased frequency with increasing occupancy. *Rhodotorula mucilaginosa* is a likely human commensal, and *Aureobasidium pullulans* was abundant in the floor dust samples. Likewise, of the 134 bacterial taxa found in most of the 8 person experiments, eight (6%) showed increased frequency with increased occupancy: *Streptophyta*, *Solibacterales*, *Corynebacterium*, *Arcobacter cryaerophilus*, *Actinomyces*, *Chroococcidiopsi*, *Oxalobacteraceae*, and *Chlorophyta*. Given that most of the detected bacterial sequences are environmental rather than skin-associated, this evidence suggests that resuspension of outdoor-derived microbes from indoor surfaces and/or from occupants’ clothing was a stronger source than direct shedding from human bodies.

We explored patterns of ecological distance between indoor and outdoor pairs; however, few patterns emerged. The absolute values of the distances between indoor and outdoor pairs were significantly less for bacteria than fungi (*t*-test, *p*<0.001). Contrary to expectations, indoor air was not observed to be more compositionally similar to outdoor air in low occupancy periods than during higher occupancy periods (*t*-test, *p*>0.05). Moreover, high occupancy periods were not found to be more compositionally similar to each other than low occupancy periods were to each other (*t*-test, *p*>0.05).

There was one fungus that stood out as an indicator of the influence of human occupancy. The most abundant fungus detected in the aggregate indoor air samples was *Battarrea stevenii*. This puffball appeared in high read abundance in two 8-person experiments and was not found in the paired outdoor air samples. The likely explanation is that a member of our research group acted as an inadvertent vector for the transport of these spores into the chamber. This research group member, who was also one of our study subjects, had previously handled specimens of *Battarrea* wearing the same sweater later worn in the chamber. In those two sampling periods, *Battarea* comprised 5% and 2% of all fungal sequences in indoor air.

### Other sources of indoor bioaerosols

Floor dust can serve as a source of bioaerosols, so we collected vacuumed floor samples of dust from the carpet. The floor dust samples, despite being collected months apart, were similar in composition to each other, and were more similar to the composition of outdoor air than of indoor air (Fig 2). Considering the fifteen most abundant taxa in the respective samples, floor dust shared only one fungus (6%) and three bacteria (20%) with the indoor and outdoor air samples (Fig 1). The mean richness of microbes in the floor dust samples was 2× or 3× higher than that of indoor air samples for fungi and bacteria, respectively, and approximately 20% of the taxa detected were specific to the floor dust samples. The fungi in the air of walking experiments were slightly more similar to the floor dust when the carpet was exposed than when it was covered (mean difference 0.85 versus 0.88, *p*-value 0.05), while there was no difference for bacteria.

The ventilation system itself could be a source of microbes, and we included a secondary set of experiments that included sampling the supply air. This effort yielded only a few data points, so we simply note the patterns. The air samples from the secondary study were similar to each other and distinct from the main study samples to varying degrees: for fungi, the secondary samples were quite different compositionally from the main study samples but for bacteria less so (Fig 2). For neither bacteria nor fungi was the ventilation system itself an obvious source of indoor microbes. That is, the taxa that were abundant in the supply air were also abundant outdoors. Those taxa that are present in the supply and indoor air but absent from outdoor air have relatively low read abundance (median read abundance of 18 sequences).

### Effect of occupancy on total particle concentrations

**Fig 3:**
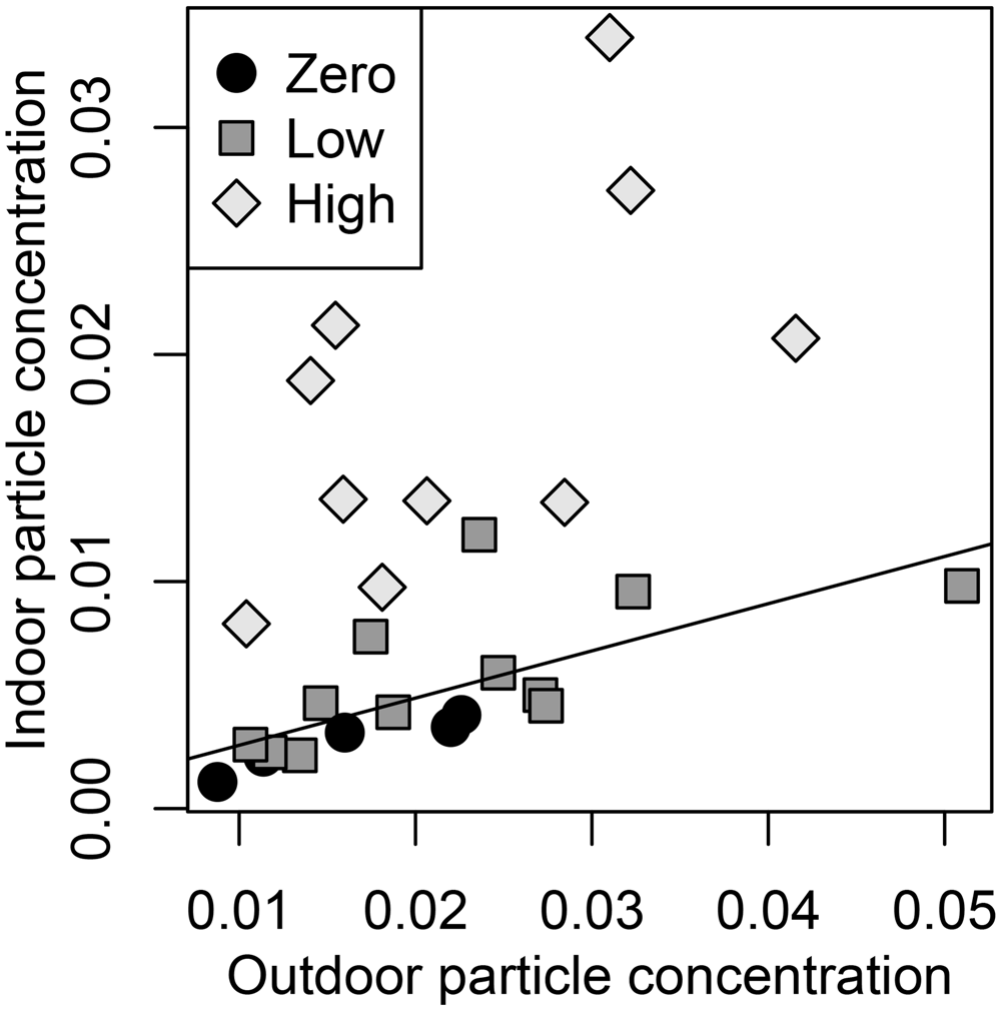
Mean concentrations of particles larger than 5 microns. Concentrations were evaluated with optical particle counters during experiments that occurred over 10 calendar days and when the carpet was exposed or covered in a plastic sheet. Values show particle numbers per cm^3^ air. Low occupancy periods had 1 or 2 people sitting, and high occupancy periods had 2 people walking or 8 people sitting. The linear regression is based on zero and low occupancy periods.

Occupants had a significant effect on total particle concentrations in the chamber air. Table 3 presents size-specific indoor/outdoor total particle number concentration ratios (I/O) under three categories of occupancy conditions: zero, low (1–2 occupants seated), and high (8 occupants seated or 2 occupants walking). In general, I/O ratios are influenced by particle removal through filtration of supply air, particle loss via deposition on chamber surfaces, and particle generation by occupants. An increase in filtration efficiency and/or deposition loss rate with particle size is evident in the data: the zero-occupancy I/O ratio declines from about 0.7 for 0.3–0.5 μm particles to 0.17–0.19 for particles larger than 5 μm. Table 3 shows that, for particles smaller than 2 μm, I/O ratios were not clearly affected by occupancy. Conversely, there was a clear impact of occupancy on larger particles that was stronger when the floor was exposed. Even for the particles most strongly impacted by occupancy (>5 μm), indoor particle concentrations with no or low occupancy loads correlate with outdoor particle concentrations, and the high occupancy load periods show elevated particle concentrations relative to this baseline level (Fig 3). The fraction of indoor particles larger than 5 μm that was attributable to occupancy (versus originating from outdoor air) was in the range 62–96% when the floor was covered and 84–87% when the floor was exposed (S4 Fig). Taken together, the low I/O ratios and the attribution of particles to sources suggest a discernible yet relatively low total particle emission rate associated with occupancy in these experiments.

**Table 3:** Particle number concentrations.

	Exposed floor					
	Lower size cut (microns)					
Occupancy	0.3–0.5	0.5–0.7	0.7–1.0	1.0–2.0	2.0–5.0	>5
Zero	0.70 (0.10)	0.57 (0.09)	0.62 (0.10)	0.64 (0.09)	0.50 (0.04)	0.17 (0.03)
Low	0.73 (0.18)	0.58 (0.25)	0.62 (0.19)	0.64 (0.13)	.48 (.04)	.28 (.12)
High	0.71 (0.08)	0.55 (0.16)	0.63 (0.14)	0.72 (0.13)	0.77 (0.14)	1.10 (0.25)
	Covered floor					
	Lower size cut (microns)					
Occupancy	0.3–0.5	0.5–0.7	0.7–1.0	1.0–2.0	2.0–5.0	>5
Zero	0.73 (0.13)	0.70 (0.13)	0.63 (0.16)	0.50 (0.13)	0.48 (0.06)	0.19 (0.02)
Low	0.78 (0.07)	0.73 (0.11)	0.66 (0.20)	0.51 (0.15)	0.46 (0.07)	0.24 (0.04)
High	0.74 (0.14)	0.69 (0.12)	0.62 (0.18)	0.49 (0.14)	0.53 (0.11)	0.74 (0.14)

Mean (and standard deviation) of indoor/outdoor particle number concentration ratios for exposed versus covered carpeted floor. Means are standard deviations based on three experiments. Low-occupancy represents 1 or 2 people sitting, and high-occupancy represents 2 people walking or 8 people sitting.

## Discussion

This study contributes to the developing picture of the effect of occupancy and occupant activity on microbial composition of indoor air. This study is one of only a few that target both fungi and bacteria, and the patterns across the two groups showed high similarity. Our results showed that in a space well ventilated with modestly filtered air and that is not too densely occupied, the occupants did not provide a strong signal of microbial DNA. We discuss these findings in the context of the specific conditions of our environmental chamber and previous work examining indoor air microbial composition.

### Effect of occupancy on indoor air microbial composition

There are several aspects of the way this chamber was operated that could contribute to a strong outdoor source relative to indoor emissions. For one, the air exchange rate was relatively high, 2.8 per hour, which is in the upper range of values measured in US office buildings and high compared to the central tendency in residences [33, 34]. Given the size of the chamber, at its highest occupancy (8 people), building standards would require a lower minimum air exchange rate of ~ 1.3 per hour [35]. Air was treated through MERV 7 filters, which offer only a modest level of filtration efficiency, although this filter quality is common for use in commercial buildings [36].

Consistent with conditions that predict a strong outdoor source in the chamber, we observed a lower value for the percentage of human-associated taxa relative to other indoor microbiome studies. In the heavily occupied setting of the New York City subway system, approximately 20% of observed taxa were those associated with human skin [37]. In settings more similar to our office environment than public transportation systems, Hospodsky et al. [10] and Qian et al. [12] reported that five human-associated bacteria taxa comprised 17% of all bacteria in their university classroom air samples while, in a separate university classroom study, Meadow et al. [9] found that on average 8% of the sequences were human associated. In our study, the value was 4%. Accordingly, the proportion of sequences that were attributable to known human commensals in indoor air relative to outdoor air was enriched by a factor of 1.3 in our study, while in another study in a classroom the enrichment factor was 3.5 [12].

Despite the relatively smaller signal of human body-associated taxa we found in our study, the qualitative results—of a strong microbial link between outdoor and indoor air—matches other current studies that have paired outdoor and indoor air samples [9, 37, 38]. Even in the presence of a higher occupancy load, the composition of bacteria in the air of the New York City subway system was most similar to outdoor air [37]. A recent investigation in the Hong Kong subway found that the subway air and outdoor air were compositionally indistinguishable [38]. Similarly, Meadow et al. [9] showed that in university classrooms in the Pacific Northwest US, indoor air largely tracked outdoor air but with time lags that depended on the type of ventilation system. This dominance of outdoor air relative to indoor occupancy as a source for bioaerosols is noteworthy, particularly relative to other studies that have compared unoccupied and occupied periods [10–12] and other studies that have sampled bacteria on indoor surfaces [1, 2].

While we did observe microbial taxa associated with the human body cavity, the most striking effect of occupancy that we observed was of occupants as potential passive transport vectors of microbes from outdoors or from other indoor locations. This human-mediated dispersal was identified by the unique signal of the puffball *Batterea* being abundant in particular experiments when one specific person was present and absent in outdoor samples. Recently it was demonstrated that clothing could act as a secondary source of particles that were previously deposited on the fabric [39, 40]. Resident behavior as vectors transporting fungal material into homes has been observed in association with food [41], fuel wood chips [42], and on clothing [41, 43]. For example, Pasanen et al. [43] report the movement of *Acremonium*, *Alternaria*, *Botrytis*, and *Chryosporium* from cowsheds into homes on the clothes of residents. And, more generally, research shows that soil resuspension and track-in can be major contributors of pollutants in house dust [44].

Although we observed this human-mediated transport with *Battarea* only, it is likely important for the transport of other—perhaps most—outdoor microbes. And, in fact, integrating results from recent studies showing that occupants have a marked effect on the (bio)mass of airborne particles [11, 12, 14] but not necessarily on the composition (here and [9]) suggests that occupants may predominately be secondary emitters of environmental microbes that are largely sourced from outdoor air. That is, the microbial composition on occupants clothing may become similar to that of the outdoor air of the surrounding area, and thus the signature of occupants on indoor air microbial composition would be obscured as originating from the outdoor supply air. In this conceptualization, an occupied room would show quantitatively higher bioaerosol levels than an unoccupied room but the composition might be similar between the two cases, i.e. substantially independent of occupancy level.

We note that the potential for resuspension of environmental microbes rather than human-associated microbes might be greater in the office-like chamber used in this study than in other settings that are consistently more heavily occupied. As a testing environment, this chamber has relatively long periods of vacancy, especially compared to classrooms or homes. The microbial signature that would accumulate on interior surfaces owing to supply by ventilation and deposition by settling could come to resemble the composition of outdoor air. For those settings that are more heavily and regularly used, the settled dust that might later be resuspended could have a stronger human signature. Resuspension in the two cases—one with a history of high occupancy and another with low occupancy loads—would provide different occupancy-associated fingerprints because of differences in the reservoir makeup. As our efforts at quantitative measures were hindered (see below), further studies will be needed to more deeply explore the microbiological nature of occupancy-associated emissions.

The single most abundant bacterial taxon in the floor dust, *Bifidobacterium adolescentis*, is a known human commensal, although it was rare in both the indoor and outdoor air samples. Curiously, we did not identify DNA from the fungus *Malassezia*, a recognized abundant human commensal [45, 46]. While *Malassezia* is highly abundant on skin, its relative abundance in environmental surveys of indoor environments varies [6, 16, 47, 48]. We did detect species and members of other genera that are known to contain human commensals, typically acting as yeasts on skin surfaces: *Trichosporon* [49], *Rhodotorula mucilaginosa* [50], *Candida*, and *Cryptococcus* [51].

The simultaneous sampling of outdoor air aided in the interpretation of our results, as it showed that taxa that were observed indoors were not necessarily sourced indoors. Interestingly, skin-associated bacterial taxa were also observed in outdoor air, a pattern also reported by Qian et al. [12], suggesting that even the outdoor air might have a human commensal signal in densely populated urban settings. Plus, there were five taxa identified as the fungus *Wallemia*, and two of those as *Wallemia sebi* specifically. *Wallemia* is typically regarded as an indoor contaminant whose presence indoors indicates growth internal to the structure [31]. Only one of these *Wallemia* taxa, identified as *Wallemiales* sp., was not observed outdoors, and it was observed in only one indoor sampling period. The remaining four *Wallemia* taxa occurred both indoors and outdoors. The occurrence outdoors indicates that occurrence indoors should not be automatically attributed to indoor growth.

### Detection limits in bioaerosol sampling

In two of our sampling efforts, biomass yields were insufficient to undertake specific analyses. The first was quantitative PCR, which we employed to determine the fungal and bacterial biomass for each sampling period. Many samples were near detection limits. Across the replicates for each sample, precision was low; that is, the variation of duplicated measurements of the same sample tended to be high. We present details on methods and results in S2 File. Quantification of biological material is an important vehicle for linking microbial composition with particle dynamics. With our sampling methods, we conclude that biomass yields were too low for robust conclusions based on qPCR. High-throughput sequencing is a more sensitive measure than qPCR and has been successfully reported for volumes of air similar to the ones we use here [38].

The second detection limit challenge we encountered was with particle collection using the National Institute for Occupational Safety and Health (NIOSH) 2-stage cyclone aerosol sampler, the BC 251 [52]. The first stage of the sampler fitted a disposable 15-mL Falcon collection tube and had a lower particle size cutoff of 3.7 μm aerodynamic diameter. The second stage employed a disposable 1.5-mL Eppendorf tube and collected particles in the size range 0.74–3.7 μm. Attempts at amplification, after extraction following the protocol detailed above, were unsuccessful from the smaller size fraction and were inconsistent from the larger size fraction. Due to these patchy results, the cyclone samples were not processed further. The results suggested that in our study environment, the volume of air sampled (0.6 m^3^) was insufficient to obtain a signal clearly above detection limits. The NIOSH cyclone sampler requires a lower flow rate vacuum pump, and successful use of this sampler in environmental settings has been based on longer sampling times [53] than we used here. Both qPCR and the NIOSH cyclone sampler rely on the extraction of DNA as the material for analysis. Although we used an extraction protocol developed for indoor environments [16] that mirrors another laboratory’s practices [10], there may be modifications in the extraction process that could increase efficiency.

### Observations on Illumina community data

Determining microbial composition with high-throughput sequencing is a powerful technique, and its utilization in indoor air research has great potential to offer important insight into bioaerosol dynamics [54, 55]. However, there are several steps in the processing pipeline that require particular attention [56], and best practices have not been agreed upon (e.g. [57]). One particular issue that we highlight here is treatment of low-abundance reads, sequences that are present at sparse levels. While some of these reads may represent true biological diversity [58], many may be spurious OTUs due to chimeric sequences, errors during PCR amplification, or imprecise OTU binning. As a quality assurance measure for our samples, we ran a mock community, which indicated that reads with fewer than 10 sequences in a sample were potentially spurious. Of the 84 taxa excluded from the mock community by this threshold, 10% were typed to the same taxonomy as the high-abundance reads, which would indicate spurious OTUs attributable to PCR error or imprecise OTU binning. The remaining low-abundance reads from the mock community were present at high abundance in other samples, which is indicative of barcode tag switching events (so called “mistagging” [59, 60]). Results we report here were robust to inclusion or exclusion of these low abundance reads, supporting the general idea that these high-throughput methods are strong for questions that center on relationships among samples (beta-diversity) but can require more caution for exploring specific questions about community membership (alpha-diversity) [61].

## Conclusions

In this environmental chamber study, during common indoor activities (sitting and walking), the strength of the primary signal from human occupants and their activities was minor in the context of the entire fungal and bacterial bioaerosol communities that derived from all sources. We conclude that even in mechanically ventilated buildings, if the air-exchange rate is high, the filter efficiency poor to moderate, and the occupancy light to moderate, the indoor air microbiology can look much like that in outdoor air. For a full integration of microbial ecology with aerosol science, practical hurdles remain to be resolved for improved detection limits and in linking particle dynamics with bioaerosol behavior. We found evidence that humans can be vectors for microbes to enter the built environment through tracking on clothing and subsequent shedding; the importance of this source contribution to the indoor microbiome may be underappreciated.

## Supporting Information

S1 Fig The controlled environment chamber. Boxed are the sampling devices: (A) inverted analytical filter cup; (B) BC251 two-stage cyclone impactor; and (C) optical particle counter.
(TIF)

S2 Fig Visualization of the relationship between samples in their bacterial composition based on the phylogenetically informed UniFrac distance metric. Principal coordinate plot showing the relationship between the community composition across samples. Compare with Fig 2 of the main text.
(TIF)

S3 Fig Observed OTU richness across the occupancy levels. Fungi are on top while bacteria are on bottom, and richness is split across indoor (left) and outdoor (right) samples.
(TIF)

S4 Fig Fraction of the mean indoor concentration of total particles larger than 5 μm that is attributable to occupancy. Results are based on particles measured during the second hour of each treatment.
(TIF)

S1 File Molecular methods. Details of the experimental conditions used to generate and process the Illumina MiSeq library.
(DOCX)

S2 File Quantitative PCR. Summary of the methods and results of quantitative PCR.
(DOCX)

S1 Table Schedule of experimental treatments. (DOCX)

## References

[1] Lax S, Smith DP, Hampton-Marcell J, Owens SM, Handley KM, Scott NM, et al Longitudinal analysis of microbial interaction between humans and the indoor environment. Science. 2014;345(6200):1048–1052. 10.1126/science.1254529 25170151 PMC4337996

[2] Meadow J, Altrichter AE, Kembel SW, Moriyama M, O'Connor TK, Womack A, et al Bacterial communities on classroom surfaces vary with human contact. Microbiome. 2014;2(1):7 10.1186/2049-2618-2-7 24602274 PMC3945812

[3] Fox A, Harley W, Feigley C, Salzberg D, Sebastian A, Larsson L. Increased levels of bacterial markers and CO_2_ in occupied school rooms. J Environ Monitor. 2003;5(2):246–252. 10.1039/B212341j 12729263

[4] Millington WM, Corden JM. Long term trends in outdoor *Aspergillus*/*Penicillium* spore concentrations in Derby, UK from 1970 to 2003 and a comparative study in 1994 and 1996 with the indoor air of two local houses. Aerobiologia. 2005;21(2):105–113. 10.1007/s10453-005-4180-1

[5] Täubel M, Rintala H, Pitkäranta M, Paulin L, Laitinen S, Pekkanen J, et al The occupant as a source of house dust bacteria. J Allergy Clin Immunol. 2009;124(4):834–840. 10.1016/j.jaci.2009.07.045 19767077

[6] Pitkäranta M, Meklin T, Hyvärinen A, Paulin L, Auvinen P, Nevalainen A, et al Analysis of fungal flora in indoor dust by ribosomal DNA sequence analysis, quantitative PCR, and culture. Appl Environ Microbiol. 2008;74(1):233–244. 10.1128/AEM.00692-07 17981947 PMC2223223

[7] Adams RI, Miletto M, Lindow SE, Taylor JW, Bruns TD. Airborne bacterial communities in residences: similarities and differences with fungi. PLoS ONE. 2014;9(3):e91283 10.1371/journal.pone.0091283 24603548 PMC3946336

[8] Hewitt KM, Gerba CP, Maxwell SL, Kelley ST. Office Space Bacterial Abundance and Diversity in Three Metropolitan Areas. PLoS ONE. 2012;7(5):e37849 10.1371/journal.pone.0037849 22666400 PMC3364274

[9] Meadow JF, Altrichter AE, Kembel SW, Kline J, Mhuireach G, Moriyama M, et al Indoor airborne bacterial communities are influenced by ventilation, occupancy, and outdoor air source. Indoor Air. 2014;24(1):41–48. 10.1111/ina.12047 23621155 PMC4285785

[10] Hospodsky D, Qian J, Nazaroff WW, Yamamoto N, Bibby K, Rismani-Yazdi H, et al Human occupancy as a source of indoor airborne bacteria. PLoS ONE. 2012;7(4):e34867 10.1371/journal.pone.0034867 22529946 PMC3329548

[11] Hospodsky D, Yamamoto N, Nazaroff WW, Miller D, Gorthala S, Peccia J. Characterizing airborne fungal and bacterial concentrations and emission rates in six occupied children’s classrooms. Indoor Air. 10.1111/ina.12172 25403276

[12] Qian J, Hospodsky D, Yamamoto N, Nazaroff WW, Peccia J. Size-resolved emission rates of airborne bacteria and fungi in an occupied classroom. Indoor Air. 2012;22(4):339–351. 10.1111/j.1600-0668.2012.00769.x 22257156 PMC3437488

[13] Yamamoto N, Hospodsky D, Dannemiller K, Nazaroff WW, Peccia J. Indoor Emissions as a Primary Source of Airborne Allergenic Fungal Particles in Classrooms. Environ Sci Technol. 2015 10.1021/es506165z 25794178

[14] Bhangar S, Adams RI, Pasut W, Huffman JA, Arens EA, Taylor JW, et al Chamber bioaerosol study: Human emissions of size-resolved fluorescent biological aerosol particles. Indoor Air. 10.1111/ina.12195 25704637

[15] Arens EA, Bauman FS, Johnston LP, Zhang H. Testing of localized ventilation systems in a new controlled environment chamber. Indoor Air. 1991;1(3):263–281. 10.1111/j.1600-0668.1991.05-13.x

[16] Amend AS, Seifert KA, Samson R, Bruns TD. Indoor fungal composition is geographically patterned and more diverse in temperate zones than in the tropics. Proc Natl Acad Sci USA. 2010;107(31):13748–13753. 10.1073/Pnas.1000454107 20616017 PMC2922287

[17] Adams RI, Miletto M, Taylor JW, Bruns TD. Dispersal in microbes: Fungi in indoor air are dominated by outdoor air and show dispersal limitation at short distances. ISME J. 2013;7:1262–1273. 10.1038/ismej.2013.28 23426013 PMC3695294

[18] Barns SM, Delwiche CF, Palmer JD, Pace NR. Perspectives on archaeal diversity, thermophily and monophyly from environmental rRNA sequences. Proc Natl Acad Sci USA. 1996;93(17):9188–9193. 8799176 10.1073/pnas.93.17.9188 PMC38617

[19] Caporaso JG, Lauber CL, Walters WA, Berg-Lyons D, Huntley J, Fierer N, et al Ultra-high-throughput microbial community analysis on the Illumina HiSeq and MiSeq platforms. ISME J. 2012;6(8):1621–1624. 10.1038/ismej.2012.8 22402401 PMC3400413

[20] Smith DP, Peay KG. Sequence depth, not PCR replication, improves ecological inference from next generation DNA sequencing. PLoS ONE. 2014;9(2):e90234 10.1371/journal.pone.0090234 24587293 PMC3938664

[21] Martin M. Cutadapt removes adapter sequences from high-throughput sequencing reads. EMBnetjournal. 2011;17(1):10–12.

[22] Bolger AM, Lohse M, Usadel B. Trimmomatic: a flexible trimmer for Illumina sequence data. Bioinformatics. 2014 10.1093/bioinformatics/btu170 PMC4103590 24695404

[23] Edgar RC. UPARSE: highly accurate OTU sequences from microbial amplicon reads. Nat Methods. 2013;10:996–998. 10.1038/nmeth.2604 23955772

[24] Heinz S, Benner C, Spann N, Bertolino E, Lin YC, Laslo P, et al Simple combinations of lineage-determining transcription factors prime *cis*-regulatory elements required for macrophage and B cell identities. Mol Cell. 2010;38(4):576–589. 10.1016/J.Molcel.2010.05.004 20513432 PMC2898526

[25] Abarenkov K, Nilsson RH, Larsson KH, Alexander IJ, Eberhardt U, Erland S, et al The UNITE database for molecular identification of fungi—recent updates and future perspectives. New Phytol. 2010;186(2):281–285. 10.1111/j.1469-8137.2009.03160.x 20409185

[26] DeSantis TZ, Hugenholtz P, Larsen N, Rojas M, Brodie EL, Keller K, et al Greengenes, a chimera-checked 16S rRNA gene database and workbench compatible with ARB. Appl Environ Microbiol. 2006;72(7):5069–5072. 10.1128/Aem.03006-05 16820507 PMC1489311

[27] Nguyen NH, Smith D, Peay K, Kennedy P. Parsing ecological signal from noise in next generation amplicon sequencing. New Phytol. 2015;205(4):1389–1393. 10.1111/nph.12923 24985885

[28] Caporaso JG, Lauber CL, Walters WA, Berg-Lyons D, Lozupone CA, Turnbaugh PJ, et al Global patterns of 16S rRNA diversity at a depth of millions of sequences per sample. Proc Natl Acad Sci USA. 2011;108 (Suppl 1):4516–4522. 10.1073/pnas.1000080107 20534432 PMC3063599

[29] R Development Core Team. R: A Language and Environment for Statistical Computing. R Foundation for Statistical Computing version 3.1.2 Vienna, Austria; 2014 Available: http://www.R-project.org. 10.1016/j.jneumeth.2014.06.019

[30] Caporaso JG, Kuczynski J, Stombaugh J, Bittinger K, Bushman FD, Costello EK, et al QIIME allows analysis of high-throughput community sequencing data. Nat Methods. 2010;7(5):335–336. 10.1038/Nmeth.F.303 20383131 PMC3156573

[31] Flannigan B, Samson RA, Miller JD, editors. Microorganisms in home and indoor work environments: Diversity, health impacts, investigation and control. 2nd ed Boca Raton: CRC Press; 2011.

[32] Lozupone C, Knight R. UniFrac: a new phylogenetic method for comparing microbial communities. Appl Environ Microbiol. 2005;71(12):8228–8235. 10.1128/Aem.71.12.8228-8235.2005 16332807 PMC1317376

[33] Persily AK, Gorfain J, Brunner G. Survey of ventilation rates in office buildings. Build Res Inf. 2006;34(5):459–466.

[34] Wu X, Apte MG, Bennett DH. Indoor particle levels in small- and medium-sized commercial buildings in California. Environ Sci Technol. 2012;46(22):12355–12363. 10.1021/es302140h 23043678

[35] ASHRAE. Ventilation: Ventilation for Acceptable Indoor Air Quality. Atlanta, GA: American Society of Heating, Refrigerating and Air Conditioning Engineers 2013 ANSI/ASHRAE Standard 62.1–2013.

[36] Bennett DH, Apte MG, Wu X, Trout A, Faulkner D, Maddalena R, et al. Indoor environmental quality and heating, ventilating, and air conditioning survey of small and medium size commercial buildings: Field study: California Energy Commission, 2011 CEC-500-2011-043.

[37] Robertson CE, Baumgartner LK, Harris JK, Peterson KL, Stevens MJ, Frank DN, et al Culture-independent analysis of aerosol microbiology in a metropolitan subway system. Appl Environ Microbiol. 2013;79(11):3485–3493. 10.1128/Aem.00331-13 23542619 PMC3648054

[38] Leung MHY, Wilkins D, Li EKT, Kong FKF, Lee PKH. Diversity and dynamics of the indoor air microbiome in an urban subway network. Appl Environ Microbiol. 2014; 10.1128/AEM.02244-14 PMC4249038 25172855

[39] McDonagh A, Byrne MA. A study of the size distribution of aerosol particles resuspended from clothing surfaces. J Aerosol Sci. 2014;75:94–103. 10.1016/J.Jaerosci.2014.05.007

[40] McDonagh A, Byrne MA. The influence of human physical activity and contaminated clothing type on particle resuspension. J Environ Radioact. 2014;127:119–126. 10.1016/J.Jenvrad.2013.10.012 24211670

[41] Lehtonen M, Reponen T, Nevalainen A. Everyday activities and variation of fungal spore concentrations in indoor air. Int Biodeterior Biodegrad. 1993;31(1):25–39.

[42] Miller JD, Schneider MH, Whitney NJ. Fungi on fuel wood chips in a home. Wood Fiber Sci. 1982;14(1):54–59.

[43] Pasanen AL, Kalliokoski P, Pasanen P, Salmi T, Tossavainen A. Fungi carried from farmers work into farm homes. Am Ind Hyg Assoc J. 1989;50(12):631–633. 2596402 10.1080/15298668991375272

[44] Layton DW, Beamer PI. Migration of contaminated soil and airborne particulates to indoor dust. Environ Sci Technol. 2009;43(21):8199–8205. 10.1021/Es9003735 19924944 PMC2782798

[45] Findley K, Oh J, Yang J, Conlan S, Deming C, Meyer JA, et al Topographic diversity of fungal and bacterial communities in human skin. Nature. 2013;498:367–370. 10.1038/nature12171 23698366 PMC3711185

[46] Oh J, Byrd AL, Deming C, Conlan S, NISC Comparative Sequencing Program, Kong HH, et al Biogeography and individuality shape function in the human skin metagenome. Nature. 2014;514(7520):59–64. 10.1038/nature13786 25279917 PMC4185404

[47] Pitkäranta M, Meklin T, Hyvärinen A, Nevalainen A, Paulin L, Auvinen P, et al Molecular profiling of fungal communities in moisture damaged buildings before and after remediation—a comparison of culture-dependent and culture-independent methods. BMC Microbiol. 2011;11(1):235. 22017920 10.1186/1471-2180-11-235 PMC3206440

[48] Adams RI, Miletto M, Taylor JW, Bruns TD. The diversity and distribution of fungi on residential surfaces. PLoS ONE. 2013;8(11):e78866 10.1371/journal.pone.0078866 24223861 PMC3815347

[49] Walsh TJ, Melcher GP, Lee JW, Pizzo PA. Infections due to Trichosporon species: new concepts in mycology, pathogenesis, diagnosis and treatment. Cur Top Med Mycol. 1993;5:79–113. 8242806

[50] Wirth F, Goldani LZ. Epidemiology of *Rhodotorula*: An emerging pathogen. Interdiscip Perspect Infect Dis. 2012;2012:Article ID 465717. 10.1155/2012/465717 PMC3469092 23091485

[51] Marples MJ. The ecology of the human skin. Springfield, Ill: Thomas; 1965.

[52] Blachere FM, Lindsley WG, Pearce TA, Anderson SE, Fisher M, Khakoo R, et al Measurement of airborne influenza virus in a hospital emergency department. Clin Infect Dis. 2009;48(4):438–440. 10.1086/596478 19133798

[53] Lindsley WG, Schmechel D, Chen BT. A two-stage cyclone using microcentrifuge tubes for personal bioaerosol sampling. J Environ Monitor. 2006;8(11):1136–1142. 10.1039/b609083d 17075620

[54] Ramos T, Stephens B. Tools to improve built environment data collection for indoor microbial ecology investigations. Build Environ. 2014;81(0):243–257. 10.1016/j.buildenv.2014.07.004

[55] Peccia J, Hospodsky D, Bibby K. New directions: A revolution in DNA sequencing now allows for the meaningful integration of biology with aerosol science. Atmos Environ. 2011;45(10):1896–1897. 10.1016/J.Atmosenv.2010.11.037

[56] Lindahl BD, Nilsson RH, Tedersoo L, Abarenkov K, Carlsen T, Kjoller R, et al Fungal community analysis by high-throughput sequencing of amplified markers—a user's guide. New Phytol. 2013;199(1):288–299. 10.1111/nph.12243 23534863 PMC3712477

[57] McMurdie PJ, Holmes S. Waste not, want not: Why rarefying microbiome data is inadmissible. PLoS Comput Biol. 2014;10(4):e1003531 10.1371/journal.pcbi.1003531 24699258 PMC3974642

[58] Huse SM, Welch DBM, Sogin ML. Sequencing errors, diversity estimates, and the rare biosphere: Workshop summary 2013 In: The science and applications of microbial genetics [Internet]. Washington, DC: The National Academies Press; [188–207].

[59] Philippe E, Lejzerowicz F, Pawlowski J. Accurate multiplexing and filtering for high-throughput amplicon-sequencing. Nucleic Acids Res. 2015 10.1093/nar/gkv107 PMC4357712 25690897

[60] Carlsen T, Aas AB, Lindner D, Vrålstad T, Schumacher T, Kauserud H. Don't make a mista(g)ke: is tag switching an overlooked source of error in amplicon pyrosequencing studies? Fungal Ecol. 2012;5(6):747–749.

[61] Ley RE, Hamady M, Lozupone C, Turnbaugh PJ, Ramey RR, Bircher JS, et al Evolution of mammals and their gut microbes. Science. 2008;320(5883):1647–1651. 10.1126/science.1155725 18497261 PMC2649005

