# Correction: Chamber Bioaerosol Study: Outdoor Air and Human Occupants as Sources of Indoor Airborne Microbes

**DOI:** 10.1371/journal.pone.0133221

**Published:** 2015-07-14

**Authors:** Rachel I. Adam, Seema Bhangar, Wilmer Pasut, Edward A. Arens, John W. Taylor, Steven E. Lindow, William W. Nazaroff, Thomas D. Bruns

There is an error in the first sentence of the Materials and Methods section. The correct sentence is: Experiments were conducted in a controlled environmental chamber (S1 Fig) designed to simulate an office room [15].
