# Supplementary figures and images for: Chamber Bioaerosol Study: Outdoor Air and Human Occupants as Sources of Indoor Airborne Microbes
Source: PLoS One. 2015 May 29;10(5):e0128022. doi: 10.1371/journal.pone.0128022 (PMC4449033; doi:10.1371/journal.pone.0128022)

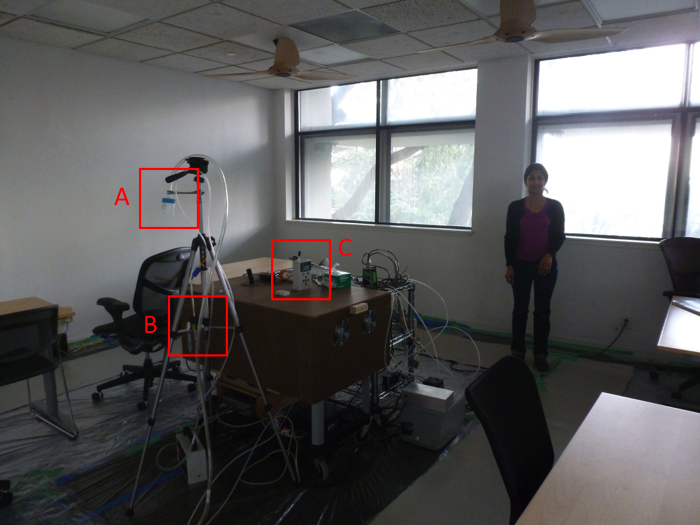

Supplement: S1 Fig — Boxed are the sampling devices: (A) inverted analytical filter cup; (B) BC251 two-stage cyclone impactor; and (C) optical particle counter. (TIF) [file pone.0128022.s001.tif]

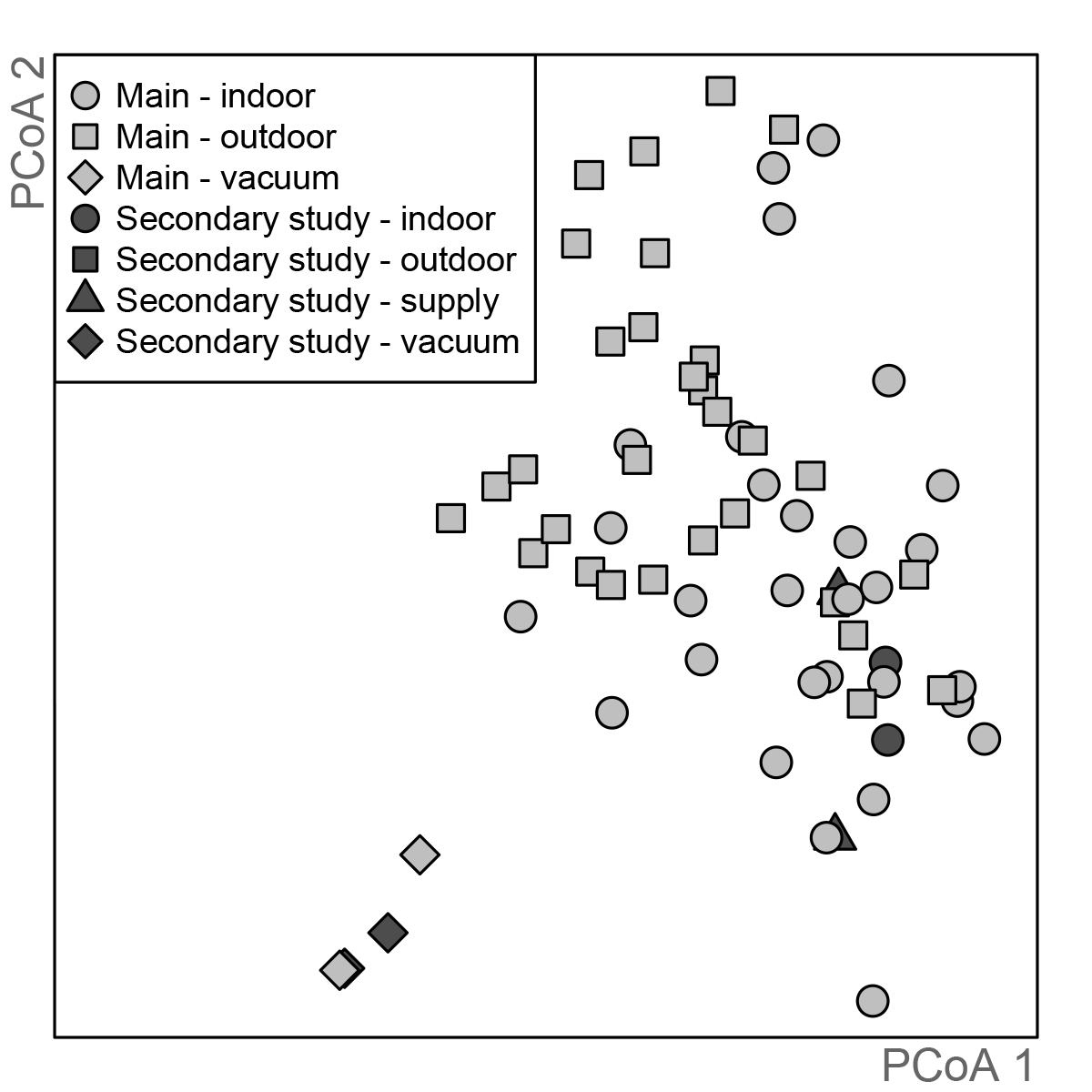

Supplement: S2 Fig — Principal coordinate plot showing the relationship between the community composition across samples. Compare with Fig 2 of the main text. (TIF) [file pone.0128022.s002.tif]

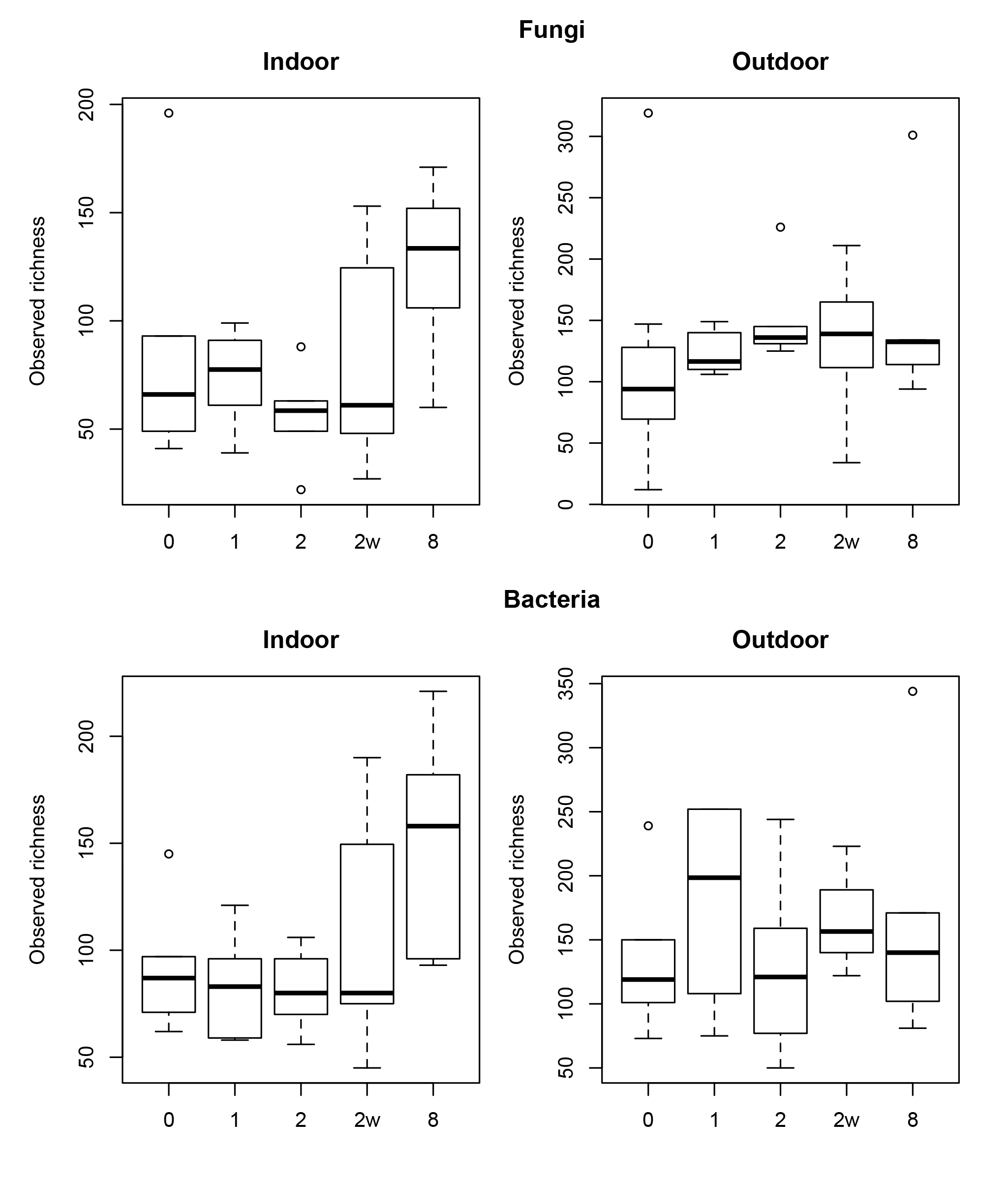

Supplement: S3 Fig — Fungi are on top while bacteria are on bottom, and richness is split across indoor (left) and outdoor (right) samples. (TIF) [file pone.0128022.s003.tif]

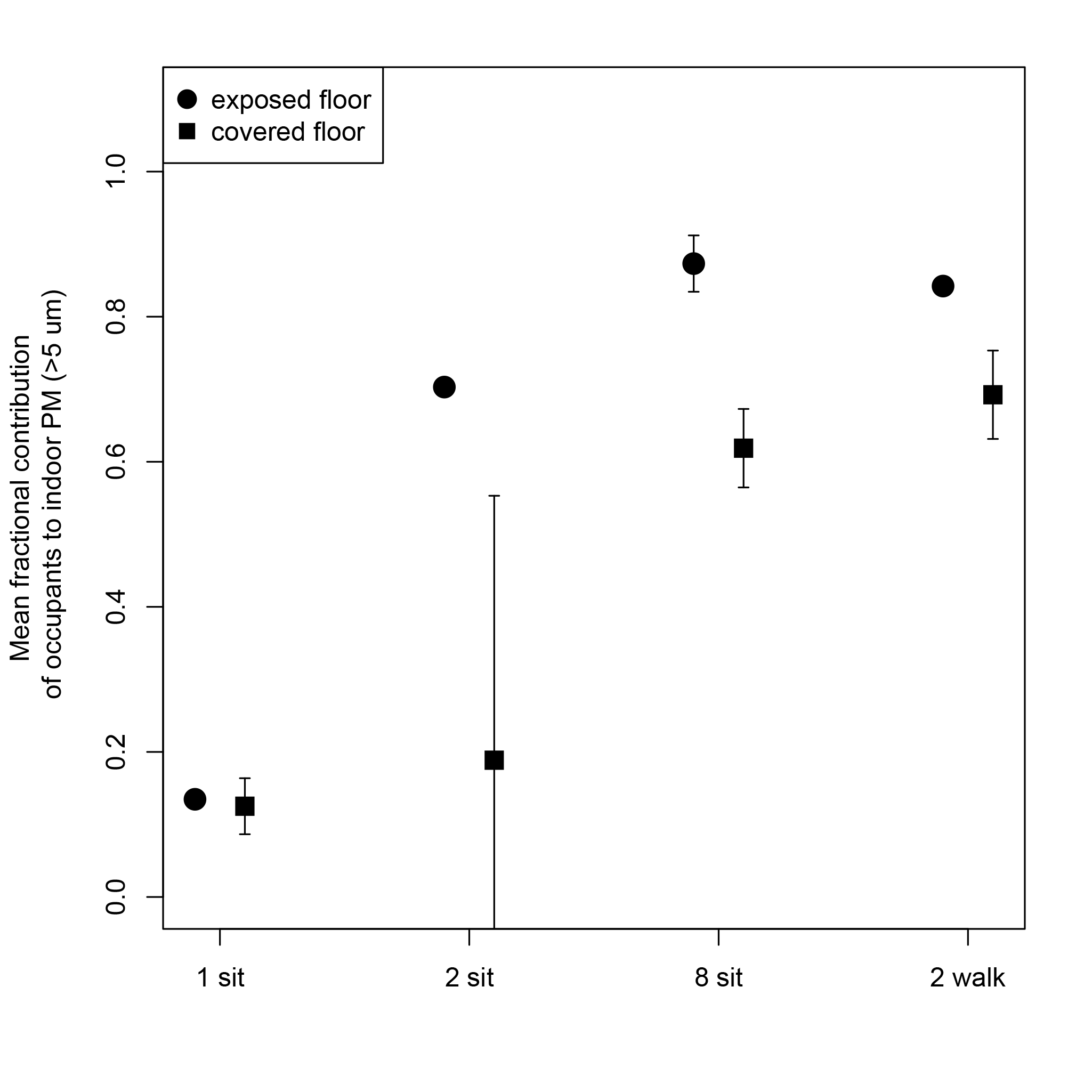

Supplement: S4 Fig — Results are based on particles measured during the second hour of each treatment. (TIF) [file pone.0128022.s004.tif]
