# Supplementary material for: Chamber Bioaerosol Study: Outdoor Air and Human Occupants as Sources of Indoor Airborne Microbes
Source: PLoS One. 2015 May 29;10(5):e0128022. doi: 10.1371/journal.pone.0128022 (PMC4449033; doi:10.1371/journal.pone.0128022)
Supplement: S1 File — Details of the experimental conditions used to generate and process the Illumina MiSeq library. (DOCX) [file pone.0128022.s005.docx]

**Text S3. Molecular methods.** Details of the experimental conditions used to generate and process the Illumina MiSeq library.

**DNA extraction**

Following [1], starting material was half of the 47-mm filter from the filter cup, cut by using flame-sterilized scissors, or 200 mg of unprocessed dust from the vacuumed floor dust sample. Samples were combined with 400 μL Miller SDS buffer, 400 μL Miller Phosphate buffer, and lysing beads, and bead beat at full speed for 1 minute. An aliquot of 450 μL of 25:24:1 phenol: chloroform: isoamyl alcohol was added and then a second round of 1 minute bead beating was done. Samples were centrifuged at 10,000 g for 5 minutes at 4 °C. Approximately 500 μL of the supernatant was isolated, and at this point the MoBio PowerSoil Kit was used for genomic DNA isolation starting with solution C4. Two rounds of 50 μL of warm C6 were used for elution.

**Library preparation**

We targeted the V4-V5 region of the bacterial 16S rRNA gene and the ITS1 region of the fungal rRNA gene. Bacterial primers were those adopted for the Earth Microbiome Project [2] while fungal primers were those recently described by Smith and Peay [3]. PCR amplification reactions contained 0.13 μL HotStarTaq, 2.5 μL 10X buffer, 0.5 μL of 2 μM dNTPs, 0.5 μL of 10 μM of the tagged forward primer, 1 μL of 5 μM of the tagged and barcoded reverse primer, 0.25 μL of 100 mg/mL bovine serum albumin (BSA), 5 μL of sample DNA, and water to 25 μL. Thermocycler conditions were as follows: denaturation at 95 °C for 5:00 followed by 30 cycles of 95 °C for 0:30, annealing for 0:30 at 51 °C for fungi and at 50 °C for bacteria, extension at 72 °C for 1:00, and finish with a final extension of 72 °C for 10:00. The PCR products were cleaned using Agencourt AMPure magnetic beads (Beckman Coulter, Brea, CA, USA) and quantified with the Qubit dsDNA HA assay kit (Invitrogen, Carlsbad, CA, USA). Cleaned products were pooled at an equimolar concentration and split across two Illumina MiSeq lanes for 250 base pair paired-end sequencing at the Stanford Functional Genomics Facility.

**Bionformatics pipeline for processing Illumina sequencing reads.** Follows the methods developed by Smith and Peay [3].

*Fungi*

We used cutadapt [4] to remove adapter sequences with quality filtering set at 20 and an error rate of 0.2 for the forward reads and 0.3 for the reverse. Trimmomatic [5] was used to identify those reads that have a pair, cut the leading and trailing 20 base pairs, and require a minimum length of 75 basepairs (bp). The reads were then merged using USEARCH [6], again with a minimum length of 75 bp and quality truncation set to 3. We then attempted a second route of pairing using USEARCH after applying homerTools [7] to trim the tailing 36 bp on the forward reads and 45 bp on the reverse reads. The two rounds of merged pairs were combined for downstream analysis following the UPARSE pipeline: filtered reads with a maximum expected error of 0.5, dereplicated sequences, sorting by read length, and then clustering into operational taxonomic units (OTUs) at the 97% similarity threshold. Resulting OTUs were checked for chimeras using uchime and were assigned taxonomic identification against the UNITE database [8], version released February 9, 2014.

*Bacteria*

Bionformatic processing of bacteria is typically more straightforward than for fungi because, while the target locus of fungi (ITS1) is of variable length, the amplicons of bacterial 16S are of uniform length. In our case, the reverse “R2” reads for bacteria exhibited extremely low quality; consequently, we proceeded with the forward “R1” reads only. Trimmomatic [5] was used to cut the leading and trailing 3 base pairs, and required a minimum length of 75 basepairs (bp). Using USEARCH, we filtered the reads with a maximum expected error or 0.5 and a minimum length of 200 bp, dereplicated sequences, sorted by read length, and then clustered into operational taxonomic units (OTUs) at 97% similarity. Resulting OTUs were checked for chimeras using uchime against the gold database and were assigned taxonomic identification against the Greengenes database [9] version released May 2013.

1. Adams RI, Miletto M, Taylor JW, Bruns TD (2013) Dispersal in microbes: Fungi in indoor air are dominated by outdoor air and show dispersal limitation at short distances. ISME J 7: 1262-1273.

2. Caporaso JG, Lauber CL, Walters WA, Berg-Lyons D, Huntley J, et al. (2012) Ultra-high-throughput microbial community analysis on the Illumina HiSeq and MiSeq platforms. ISME J 6: 1621-1624.

3. Smith DP, Peay KG (2014) Sequence depth, not PCR replication, improves ecological inference from next generation DNA sequencing. PLoS ONE 9: e90234.

4. Martin M (2011) Cutadapt removes adapter sequences from high-throughput sequencing reads. EMBnetjournal 17: 10-12.

5. Bolger AM, Lohse M, Usadel B (2014) Trimmomatic: a flexible trimmer for Illumina sequence data. Bioinformatics doi: 10.1093/bioinformatics/btu170.

6. Edgar RC (2013) UPARSE: highly accurate OTU sequences from microbial amplicon reads. Nat Methods 10: 996–998.

7. Heinz S, Benner C, Spann N, Bertolino E, Lin YC, et al. (2010) Simple combinations of lineage-determining transcription factors prime *cis*-regulatory elements required for macrophage and B cell identities. Mol Cell 38: 576-589.

8. Abarenkov K, Nilsson RH, Larsson KH, Alexander IJ, Eberhardt U, et al. (2010) The UNITE database for molecular identification of fungi — recent updates and future perspectives. New Phytol 186: 281-285.

9. DeSantis TZ, Hugenholtz P, Larsen N, Rojas M, Brodie EL, et al. (2006) Greengenes, a chimera-checked 16S rRNA gene database and workbench compatible with ARB. Appl Environ Microbiol 72: 5069-5072.
