# Supplementary material for: Chamber Bioaerosol Study: Outdoor Air and Human Occupants as Sources of Indoor Airborne Microbes
Source: PLoS One. 2015 May 29;10(5):e0128022. doi: 10.1371/journal.pone.0128022 (PMC4449033; doi:10.1371/journal.pone.0128022)
Supplement: S2 File — Summary of the methods and results of quantitative PCR. (DOCX) [file pone.0128022.s006.docx]

**Text S2. Quantitative PCR** Summary of the methods and results of quantitative PCR

To assess the total bacterial and fungal biomass in air samples, we employed quantitative PCR. Standard curves were generated using extracted DNA from one million each of *Pseudomonas syringae* cells and *Penicillium purpurogenum* spores for genome equivalents for bacteria and fungi, respectively. For bacterial amplification we used primers 27F [1]/518R [2] and for fungal amplification we used FF2/FR1 [3]. Both primer pairs target a locus of nearly invariant length. We used specific PCR conditions described previously [4]. To confirm that inhibitory effects in airborne samples were not impeding amplification, we spiked a subset of samples with a known quantity of spore equivalents, and no inhibition was observed. All qPCR reactions were replicated three times per sample.

Measurements using qPCR yielded mixed results. Bacterial estimates ranged from ~ 1,200 to 22,000 (median: 5,300) cell equivalents/m^3^ air, while fungal estimates were generally lower with a range of ~ 60 – 16,800 (median: 650) genome equivalents/m^3^ air. (See table below.) The precision of the three replicates for any given sample was low; that is, the variation of duplicated measurements of the same sample tended to be high. Specifically, the average coefficient of variation (defined as the ratio of the standard deviation to the mean) was 45% for the bacterial replicates and 51% for the fungal samples. General trends in indoor/outdoor bacterial and fungal ratios showed some evidence of a higher I/O ratio under high occupancy experiments; however, results were highly variable. (See figure below.)

The average indoor bacteria level was somewhat lower than the average outdoor level. Average outdoor levels were similar between the “carpet exposed” and “carpet covered” periods, averaging 7300 and 8000 cell equivalents/m^3^ air, respectively. The mean indoor levels with carpet exposed was 6800 cell equivalents/m^3^ air, somewhat higher than the 4700 cell equivalents/m^3^ air average for carpet covered. Surprisingly, we did not observe any clear systematic variability with occupancy or occupant activities.

For fungi, outdoor levels were substantially higher during the “carpet exposed” than during “carpet covered” period: 4300 versus 770 genome equivalents per m^3^ air, respectively. The indoor levels averaged 930 genome equivalents per m^3^ air when the carpet was exposed and declined to 630 genome equivalents per m^3^ air when the carpet was covered. (In computing these averages, data were only considered when paired indoor and outdoor results were available.)

Table of quantitative PCR results. Results are genome equivalents/m^3^ air, and include the mean, standard deviation, and coefficient of variation. See Table S1 for experimental conditions of each of numbers.

|  | **Bacteria** | | | | **Fungi** | | | |
| --- | --- | --- | --- | --- | --- | --- | --- | --- |
|  | **Indoor** | | **Outdoor** | | **Indoor** | | **Outdoor** | |
| **#** | **Mean** | **SD** | **Mean** | **SD** | **Mean** | **SD** | **Mean** | **SD** |
| 1 | 7841 | 516 | 13208 | 2431 | 1208 | 521 | 16528 | 8237 |
| 2 | 6633 | 2284 | 6140 | 1170 | 897 | 538 | 1080 | 370 |
| 3 | 7333 | 5706 | 4153 | 1054 | 2024 | 2091 | 1653 | 369 |
| 4 | 6312 | 962 | 22156 | 1838 | 2085 | 113 | 16800 | 2845 |
| 5 | 5467 | 1776 | 16136 | 7266 | -- | -- | 2748 | 1193 |
| 6 | 6656 | 1625 | 6047 | 2409 | -- | -- | 1025 | 69 |
| 7 | 4072 | 3587 | 5297 | 2585 | 179 | 83 | 645 | 80 |
| 8 | 3743 | 574 | 2940 | 773 | -- | -- | 535 | 37 |
| 9 | 5507 | 1872 | 4931 | 1005 | 697 | 749 | 1516 | 1608 |
| 10 | 13992 | 4482 | 2097 | 417 | 1417 | 615 | 320 | 202 |
| 11 | 1551 | 1106 | 3355 | 1409 | -- | -- | -- | -- |
| 12 | 3080 | 277 | 2347 | 2277 | 323 | 136 | 1816 | 1537 |
| 13 | 9987 | 5884 | 9029 | 8983 | -- | -- | 144 | 97 |
| 14 | 3545 | 1071 | -- | -- | 175 | 94 | 1040 | 871 |
| 15 | 13109 | 9137 | 4631 | 1482 | 265 | 83 | 1253 | 215 |
| 16 | 5800 | 6508 | 3163 | 382 | 295 | 288 | 356 | 144 |
| 17 | 9309 | 3157 | 3773 | 1205 | 265 | 25 | 905 | 1000 |
| 18 | 4051 | 1661 | 5417 | 1028 | -- | -- | 1733 | 541 |
| 19 | 4609 | 2085 | 2711 | 1353 | 352 | 406 | 352 | 181 |
| 20 | 3869 | 1307 | 20889 | 14632 | 615 | 42 | 535 | 252 |
| 21 | 3028 | 1196 | 3564 | 1879 | 631 | 365 | 653 | 299 |
| 22 | 6684 | 5923 | 15568 | 22323 | -- | -- | 256 | 96 |
| 23 | 1244 | 551 | 2391 | 855 | 1264 | 37 | 359 | 42 |
| 24 | 4173 | 3434 | 1977 | 212 | 503 | 801 | 1523 | 567 |
| 25 | 2431 | 1193 | 18321 | 16832 | -- | -- | 612 | 188 |
| 26 | 7297 | 4732 | 9583 | 2733 | 145 | 65 | 1927 | 594 |
| 27 | 5473 | 1231 | 8688 | 2147 | 1976 | 50 | 931 | 87 |
| 28 | 2599 | 741 | 7905 | 6971 | 400 | 646 | 395 | 200 |
| 29 | 5789 | 2201 | 5193 | 1922 | 925 | 918 | 747 | 609 |
| 30 | 4123 | 1369 | 11220 | 2461 | 195 | 81 | 559 | 400 |
| Blank | 2372 | 1370 |  |  | 63 | 17 |  |  |

Figure showing indoor/outdoor ratios of genome equivalents across the occupancy levels for bacteria and fungi.

1. Jiang H, Dong H, Zhang G, Yu B, Chapman LR, et al. (2006) Microbial diversity in water and sediment of Lake Chaka, an athalassohaline lake in northwestern China. Appl Environ Microbiol 72: 3832-3845.

2. Muyzer G, de Waal EC, Uitterlinden AG (1993) Profiling of complex microbial populations by denaturing gradient gel electrophoresis analysis of polymerase chain reaction-amplified genes coding for 16S rRNA. Appl Environ Microbiol 59: 695-700.

3. Zhou G, Whong WZ, Ong T, Chen B (2000) Development of a fungus-specific PCR assay for detecting low-level fungi in an indoor environment. Mol Cell Probes 14: 339-348.

4. Adams RI, Amend A, Taylor JW, Bruns TD (2013) A unique signal distorts the perception of species richness and composition in high-throughput sequencing surveys of microbial communities: a case study of fungi in indoor dust. Microb Ecol 66: 735-741.
