# Supplementary material for: Chamber Bioaerosol Study: Outdoor Air and Human Occupants as Sources of Indoor Airborne Microbes
Source: PLoS One. 2015 May 29;10(5):e0128022. doi: 10.1371/journal.pone.0128022 (PMC4449033; doi:10.1371/journal.pone.0128022)
Supplement: S1 Table — (DOCX) [file pone.0128022.s007.docx]

T**able S2. Schedule of experimental treatments.**

| **Number** | **Date** | **Time** | **Activity** | **Occupancy** | **Floor** |
| --- | --- | --- | --- | --- | --- |
| 1 | 12/09/13 | 10:00-12:00 | sit | 2 | exposed |
| 2 | 12/09/13 | 13:00-15:00 | sit | 6 | exposed |
| 3 | 12/09/13 | 16:00-18:00 | unoccupied | 0 | exposed |
| 4 | 12/10/13 | 10:00-12:00 | sit | 8 | exposed |
| 5 | 12/10/13 | 13:00-15:00 | walk | 2 | exposed |
| 6 | 12/10/13 | 16:00-18:00 | sit | 1 | exposed |
| 7 | 12/11/13 | 10:00-12:00 | unoccupied | 0 | exposed |
| 8 | 12/11/13 | 13:00-15:00 | sit | 8 | exposed |
| 9 | 12/11/13 | 16:00-18:00 | sit | 2 | exposed |
| 10 | 12/12/13 | 10:00-12:00 | walk | 2 | exposed |
| 11 | 12/12/13 | 13:00-15:00 | sit | 2 | exposed |
| 12 | 12/12/13 | 16:00-18:00 | sit | 1 | exposed |
| 13 | 12/13/13 | 10:00-12:00 | sit | 1 | exposed |
| 14 | 12/13/13 | 13:00-15:00 | walk | 2 | exposed |
| 15 | 12/13/13 | 16:00-18:00 | unoccupied | 0 | exposed |
| 16 | 12/16/13 | 10:00-12:00 | sit | 2 | covered |
| 17 | 12/16/13 | 13:00-15:00 | sit | 8 | covered |
| 18 | 12/16/13 | 16:00-18:00 | unoccupied | 0 | covered |
| 19 | 12/17/13 | 10:00-12:00 | sit | 8 | covered |
| 20 | 12/17/13 | 13:00-15:00 | sit | 1 | covered |
| 21 | 12/17/13 | 16:00-18:00 | walk | 2 | covered |
| 22 | 12/18/13 | 10:00-12:00 | unoccupied | 0 | covered |
| 23 | 12/18/13 | 13:00-15:00 | sit | 8 | covered |
| 24 | 12/18/13 | 16:00-18:00 | sit | 2 | covered |
| 25 | 12/19/13 | 10:00-12:00 | walk | 2 | covered |
| 26 | 12/19/13 | 13:00-15:00 | sit | 2 | covered |
| 27 | 12/19/13 | 16:00-18:00 | sit | 1 | covered |
| 28 | 12/20/13 | 10:00-12:00 | sit | 1 | covered |
| 29 | 12/20/13 | 13:00-15:00 | walk | 2 | covered |
| 30 | 12/20/13 | 16:00-18:00 | unoccupied | 0 | covered |
| 31 | 06/20/14 | 10:00-12:00 | walk | 2 | exposed |
| 32 | 06/20/14 | 13:00-16:00 | unoccupied | 0 | exposed |
